# Characterization of 3D-Printed Moulds for Soft Lithography of Millifluidic Devices

**DOI:** 10.3390/mi9030116

**Published:** 2018-03-08

**Authors:** Nurul Mohd Fuad, Megan Carve, Jan Kaslin, Donald Wlodkowic

**Affiliations:** 1 School of Science, RMIT University, Melbourne, VIC 3083, Australia; nfua256@aucklanduni.ac.nz (N.M.F.); s3437621@student.rmit.edu.au (M.C.); 2 Australian Regenerative Medicine Institute, Monash University, Clayton, VIC 3800, Australia; jan.kaslin@monash.edu; 3 Centre for Additive Manufacturing, School of Engineering, RMIT University, Melbourne, VIC 3083, Australia

**Keywords:** stereolithography, material jetting, soft lithography, Lab-on-a-Chip, millifluidic, biodevices, biotests, polydimethylsiloxane

## Abstract

Increased demand for inexpensive and rapid prototyping methods for micro- and millifluidic lab-on-a-chip (LOC) devices has stimulated considerable interest in alternative cost-effective fabrication techniques. Additive manufacturing (AM)—also called three-dimensional (3D) printing—provides an attractive alternative to conventional fabrication techniques. AM has been used to produce LOC master moulds from which positive replicas are made using soft-lithography and a biocompatible elastomer, poly(dimethylsiloxane) (PDMS). Here we characterize moulds made using two AM methods—stereolithography (SLA) and material-jetting (MJ)—and the positive replicas produced by soft lithography and PDMS moulding. The results showed that SLA, more than MJ, produced finer part resolution and finer tuning of feature geometry. Furthermore, as assessed by zebrafish (*Danio rerio*) biotoxicity tests, there was no toxicity observed in SLA and MJ moulded PDMS replicas. We conclude that SLA, utilizing commercially available printers and resins, combined with PDMS soft-lithography, is a simple and easily accessible technique that lends its self particularly well to the fabrication of biocompatible millifluidic devices, highly suited to the in-situ analysis of small model organisms.

## 1. Introduction

Lab-on-a-chip (LOC) devices permit the manipulation of extremely small volumes of fluids and regents within networks of miniaturized channels, and are being readily adopted in the fields of biomedical and ecological testing [1,2,3,4,5,6,7]. Consequently, interest in the development of superior rapid and inexpensive prototyping and fabrication techniques has increased.

Traditionally, LOC devices have been prototyped and produced by methods such as laser cutting, micro milling, or soft lithography [5,8,9]. However, these techniques often require multiple stages of assembly, a high degree of technical expertise, and the use of clean room facilities. In the case of laser cutting, low resolution and heat-related substratum deformations may occur.

Additive manufacturing (AM) techniques such as stereolithography (SLA) and material jetting (MJ) provide an elegant and practical alternative LOC fabrication method [2,3,4,10]. By using computer-assisted design (CAD), SLA and MJ produce a solid monolithic three-dimensional (3D) object by the successive polymerization of a light-sensitive resin. The produced part requires minimal post-processing. The multiple stages of assembly and the high degree of technical proficiency required by conventional LOC manufacturing techniques is thus avoided. AM has been used to produce optically transparent LOC devices with integrated fluidic interconnects and functional elements such as pumps and valves [11,12,13,14].

The sub-millimeter resolution achieved by the market-dominant SLA and MJ machines makes them highly suited to application in millifluidic LOC device prototyping and production. SLA machines such as the FORM 2 and ProJet 7000 HD, are capable of printing layers as thin as ≥25 μm, and MJ machines such as the Objet J750 can print layers as thin as ≥14 μm. The minimum feature size achievable by SLA and MJ is dependent on accuracy, z-axis layer thickness, and laser-spot (SLA) or droplet (MJ) size. For SLA machines, features may be produced as small as ∼70 μm, and for MJ, ∼100 μm [3,14,15].

Millifluidic LOC devices typically require designs customized to the selected model metazoan organism which are typically ≤50 mm in size [10]. Consequently, LOC geometric features which are often required include channel cross-section dimensions ranging from 250–2000 μm and features with high aspect ratios (width-to-height). In general, millifluidic LOC devices are difficult to rapidly prototype, customize, and fabricate using traditional techniques and materials.

Biocompatibility of the LOC material is critical [10,16,17]. The material used in SLA and MJ is a liquid light-sensitive resin composed of a polymer–photoinitiator system, and auxiliary compounds for tuning material mechanical and aesthetic properties [18]. Companies recommend using their proprietary resins in their AM machines to optimize final print resolution and mechanical properties. However, studies have identified that quantities of toxic compounds may leach from proprietary AM materials, reviewed in [19]. For example, Zhu et al. [9] showed that leachate from several commonly available AM materials were toxic to a variety of model organisms, with exposure having both sublethal and lethal affects. Tsuda et al. [20] found that the leaching of unknown compounds from materials can unpredictably affect test organisms, and may compromise the outcomes of biological tests.

In some respects, the results found by Zhu et al. [9], and similar studies conducted by Oskui et al. [21], Alifui-Segbaya [22], and Macdonald et al. [17], were unexpected because some of the materials tested (e.g., VisiJet SL Clear) have been awarded a United States Pharmacopoeia (USP) Class VI certification [23]. However, it is important to note that this certification is highly dependent on strict adherence to post-processing cleaning procedures.

Nonetheless, these studies highlight a potential obstacle in the use of proprietary AM resins for fabricating LOC devices intended for biological applications. Since advances in AM LOC devices can enable a growing number of applications [6], ongoing research aims to develop the capability of AM systems capable of using biologically compatible substrata. Several proprietary resins are reported to be biocompatible, including the E-Shell series (Envision Tec. Inc., Dearborn, MI, USA). E-shell has been successfully applied in micro-needle fabrication with SLA-based two-photon polymerization systems [24,25].

Custom-designed open-source resins, primarily consisting of poly(ethylene glycol) diacrylate (PEG-DA), are also reported to be biocompatible [10,26,27,28]. Bhattacharjee et al. [10] used PEG-DA to fabricate biocompatible petri dishes. After printing, the petri dishes were rendered biocompatible by post-curing under ultraviolet light, followed by 24 h extraction in water to remove potential toxic compounds (i.e., uncreated monomers and photoinitiators). Rogers et al. [13] used a B9 Creator SLA printer and a PEG-DA custom resin to manufacture microfluidic valves and channels designed to be as narrow as 350 μm in diameter. Using the the AM technique of material extrusion, Tsuda et al. [20] created biocompatible LOC devices printed in thermoplastic materials, rendered biocompatible by post-treatment with oxygen plasma bonding of a silicone-based polymer.

Those involved in the design and application of AM for LOC in vitro bioassays should exercise caution in their approach, and aim to avoid the use of potentially toxic materials which may leach from AM-printed parts in aqueous media. Compared to the direct AM of LOC devices, transfer moulding has been explored as a more rapid and economic manufacturing method [29]. Transfer moulding involves the AM of moulds which are used to cast a relief in a material which has the desired properties, such as biocompatible poly(dimethylsiloxane) (PDMS). For example, Glick et al. [29] used transfer moulding to produce a biocompatible LOC with geometrically complex components and rounded channels as narrow as 100 μm. In this example, the ProJet 3000 SLA printer was used to produce the moulds, and PDMS was used for casting.

The main motivation of our work was to demonstrate an easily and widely accessible user-friendly and inexpensive fabrication method using “off-the-shelf” resins and commercially available AM printers to produce biocompatible millifluidic systems for application in bioengineering, biomedical and toxicological fields, and for use in laboratories that may or may not have direct access to AM facilities. This method utilizes standard technology and materials and requires minimum infrastructure investments, thus reducing the entry barrier to this technology when compared to conventional techniques.

In this work, we use two different AM methods—SLA (ProJet 7000 HD) and MJ (Objet350)—to produce moulds for casting PDMS, and compare these AM methods for application in PDMS transfer moulding for the production of biocompatible millifluidic LOC devices. We characterize both the moulds and PDMS replicas, and assess the toxicity of PDMS replicas by using zebrafish (*Danio rerio*) toxicity tests.

## 2. Materials and Methods

### 2.1. Metrology

Scanning electron micrographs were taken using a Quanta 200 SEM (FEI Inc., Hillsboro, OR, USA) operating at 20.0 kV and 5.0 Spot size in high vacuum. Samples were coated with Au/Pd using an in-house sputter coater operating at 15 mA for 2 min [9]. Optical profilometry was performed using a ContourGT-I 3D Optical Microscope (Bruker Daltonics Inc., Billerica, MA, USA) with stitching enabled, as described by Zhu et al. [9]. Images were analysed with Image J.

### 2.2. Additive Manufacturing

AM prototypes were designed using SolidWorks 2011 (Dassault Systems SolidWorks Corp., Waltham, MA, USA). The CAD was exported as a .stl file and processed by the AM machine software packages. SLA was performed with ProJet 7000 HD in VisiJet SL Clear resin (3D Systems Inc., Rock Hill, SC, USA), and MJ performed with Objet350 Connex, in VeroClear resin (Stratasys Inc., Eden Prairie, MN, USA) and as described earlier [9,30].

### 2.3. Soft-Lithography

Replica moulding was performed in PDMS (Sylgard 184; DowCorning Corp, Midland, MI, USA) according to a standard protocol as described earlier [31]. Briefly, the PDMS was mixed at a 10:1 weight-to-weight (w/w) ratio of elastomer base to curing agent, then degassed at 40 Torr to remove any residual air bubbles. PDMS was then poured on master moulds to achieve approximately 5 mm thickness and cured thermally at 75 ^∘^C for up to 1 h.

### 2.4. Non-Contact Laser Machining

Controls were fabricated using infra-red computer numerical control (CNC) laser machining of poly(methyl methacrylate) (PMMA) substrate by the methods described by Khoshmanesh et al. [32] and Akagi et al. [31]. The laser cutting system achieved *x*–*y* accuracy of up to 5 μm.

### 2.5. Toxicological Profiling

The OECD 236 Fish Embryo Acute Toxicity (FET) Assay [33] and fish behavioural toxicity test [9,30] were employed to assess toxicity as described previously [9,30]. Five days post-fertilisation (DPF) zebrafish (*Danio rerio*) embryos were used in all tests. Animals were treated according to Monash University Ethics Committee regulations and protocols.

## 3. Results and Discussion

A combination of AM negative relief moulds and soft lithography were used to produce a biocompatible millifluidic LOC. Preliminary validation experiments showed that this approach is highly suited to the design, prototyping, and production of LOC with cages designed to mirror the shape of the biological specimen.

The reproduction quality of geometric features of the printed moulds made by two different AM techniques was compared, and PDMS replicas were assessed using SEM and quantitative 3D topographic surface mapping using optical profilometry. The *x*–*y* and *z* resolution of the printed moulds were also evaluated and compared. Of the two techniques, SLA was found most suitable for this application.

**Figure 1:**
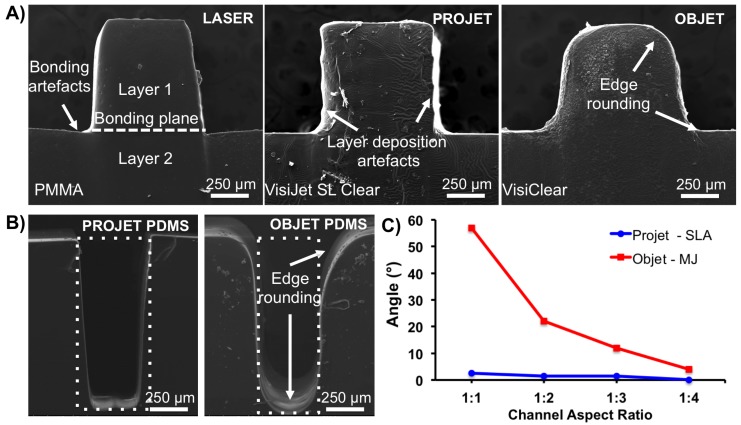
Physical characteristics of moulds fabricated using additive manufacturing processes: (**A**) SEM image of the cross-section of millifluidic positive relief pattern made with infrared laser cutting (and subsequent thermal bonding to obtain a complete mould), ProJet 7000 HD (in VisiJet SL Clear resin), and Objet350 Connex (in VeroClear resin). (**B**) Deviation from designed feature geometry of high aspect ratio poly(dimethylsiloxane) (PDMS) replicas. Dotted boxes represent the designed geometry superimposed on SEM images of PDMS channel cross-sections. (**C**) Quantitative analysis of AM moulds. Angle parameter denotes an average deviation from the designed geometry measured top plane edges of positive relief moulds. Measurements were performed on cross-sectional views obtained using SEM. Measurements were made with ImageJ software (*n* = 20).

**Figure 2:**
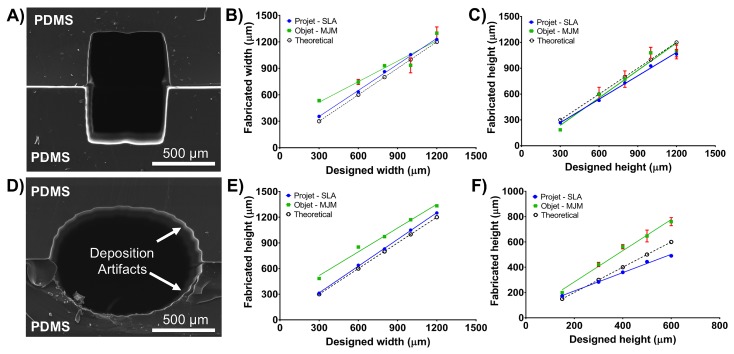
Physical characteristics of millifluidic channel replicas obtained from stereolithography (SLA; ProJet) or material jetting (MJ; Objet350 Connex) printed moulds: (**A**) SEM image of the channel with rectangular cross-section formed by moulding PDMS on a positive relief pattern made with ProJet 7000 HD (in VisiJet SL Clear resin). The channel was obtained from two identical and sandwiched PDMS replicas; (**B**) Relationship between the designed and actual width parameter of rectangular channels fabricated using SLA or MJ; (**C**) The relationship between the designed and actual height parameter of rectangular channels fabricated using SLA or MJ; (**D**) SEM micrographs of the channel with circular cross-section formed by moulding PDMS on a positive relief pattern made with ProJet 7000 HD in VisiJet SL Clear resin; (**E**) Relationship between the designed and actual feature width (defined here as a diameter in the centre plane) of circular channels fabricated using SLA or MJ; and (**F**) The relationship between the designed and actual height parameter (defined here as a diameter in the vertical plane) of circular channels fabricated using SLA or MJ. Measurements using ImageJ software were performed on cross-sectional views obtained using SEM (*n* = 20).

The two AM machines used in this study were the Objet350 Connex (MJ) and ProJet 7000 HD (SLA), and both are claimed to have ”ultra-high definition” capabilities. Positive relief test structures (moulds) were fabricated using the ProJet 7000 HD in VisiJet SL Clear resin, and the Objet350 Connex in VeroClear resin. Accuracy of the moulds was evaluated by comparing the fabricated dimensions to the dimensions in the CAD. There was variability between SLA and MJ in terms of accuracy (Figure 1 and Figure 2).

In general, we found that the smallest feature reliably produced by both machines was ∼300 μm in width and height. Features such as rectangular channel geometries with straight sidewalls fabricated by the ProJet 7000 HD more closely matched the theoretical CAD design than the features fabricated by the Objet350 Connex.

For positive relief test structures fabricated by the ProJet 7000 HD, deviation from the designed geometry was typically <10%, irrespective of feature dimension, and vertical walls exhibited an angular skew in the vertical plane of 6–9${}^{\circ }$ compared to the design parameters (Figure 1). In contrast, those fabricated by the Objet350 Connex failed to reproduce straight edges and corners. Considerable edge rounding—up to 60${}^{\circ }$—occurred at the bottom and top planes of the channels. Rounding was more pronounced when fabricating features ≤600 μm in height, which resulted in deviations from the designed geometry >40%.

The Objet350 Connex tended to exceed the designed channel width; for example, when printing features designed to be smaller than 400 μm, the printed object feature was ∼650 μm (±25μm) (Figure 2). Accuracy improved when fabricating channels designed to be larger than 900 μm in width. While the printer achieved excellent results in terms of height deposition, significant edge rounding artefacts prevented the printed channels having a rectangular shape as specified by design. Furthermore, the Objet350 Connex could not reproduce semicircular geometries reliably, and deviations from the designed geometry exceeded 40%.

We explored the fabrication of millifluidic channels with both rectangular and circular cross-sections (Figure 2, and Figure S1). When fabricating channels with both rectangular and circular cross-sections from two sandwiched PDMS replicas, the ProJet 7000 HD achieved the most consistency with theoretical design parameters. For instance, a channel with a designed geometry of 500 × 500 μm, width and height, was reproduced with dimensions of 520 ± 6 μm × 484 ± 5 μm (Figure 2). This corresponded to average deviations from a desired CAD geometry in *x* and *y*-axis of ∼5%. Overall, the ProJet 7000 HD tended to exceed channel width by 5–7%. The ProJet 7000 HD also failed to match the CAD file with respect to height. The degree of inaccuracy varied depending on the designed height. Precision decreased for designed rectangular channel heights of 1000 μm and for circular cross-section heights of 400 μm (Figure 2).

The control method—infra-red laser cutting—was unable to fabricate an optically transparent positive relief pattern with a channel width <300 μm. Achievable laser *x*–*y* accuracy was up to 5 μm. The vertical walls exhibited an angular skew in the vertical plane of ∼8${}^{\circ }$ compared to the specified design parameters (Figure 1). This is likely due to significant thermal deformation and melting damage of PMMA sheets (data not shown). These results show that these geometries are not easily fabricated by laser micromachining or photolithography methods.

**Figure 3:**
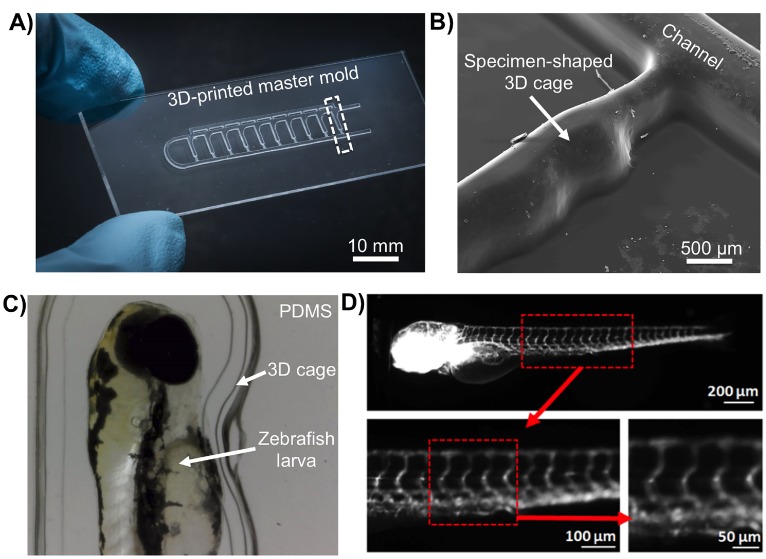
Fabrication of miniaturized 3D cages that mirror the shape of the biological specimens such as the larval stages of zebrafish (*Danio rerio*) using SLA moulds: (**A**) Microphotograph of a positive relief pattern fabricated using a ProJet 7000 HD in VisiJet SL Clear resin. The mould is the size of a standard microscope slide 25 mm × 75 mm. The dashed box denotes a single 3D cage; (**B**) Topographic surface analysis of a single 3D cage (marked as dashed box in (A) using scanning electron microscopy (SEM); (**C**) *D. rerio* larvae immobilized inside a PDMS cage, made from an SLA mould and that mirrors the organisms shape; and (**D**) High-resolution fluorescence imaging of a transgenic Fli1a:EGFP *D. rerio* larva showing green fluorescent protein (GFP)-expressing vasculature. Larger and smaller vessels are clearly distinguishable in the trunk region (boxed area, red outline).

A considerable advantage of using SLA to fabricate LOC moulds is that it permits fine control over complex topologies without any additional cost or complex protocols. Accordingly, we explored the capability of fabricating miniaturized 3D-cages that mirrored the shape of the selected biological specimen, larval zebrafish (*D. rerio*) (Figure 3). Such 3D geometries are typically difficult to achieve using conventional methods such as laser micromachining or photolithography. In addition, these methods require multiple stages of assembly, multiple masks, repeated coatings, photoresist re-flow, development, and alignment procedures.

Soft-lithography moulds the size of a standard microscope slide (25 mm × 75 mm; Figure 3) were fabricated using SLA process in under 40 min, and cost ∼US$2 per template. In comparison, laser machining soft-lithography moulds took ∼5 min, and cost ∼US$0.2 per mould. However, the control method was more labour intensive than SLA. It required the cutting of independent PMMA layers, subsequent alignment for assembly in 3D structures, and then thermal bonding for >60 min. Whereas AM required transferring the CAD to the AM software.

PDMS cured well on the VisiJet SL Clear moulds and VisiJet SL Clear moulds were geometrically stable during the PDMS curing process at 70 ${}^{\circ }$C for 1 h. In addition, the releasing of PDMS replicas did not require any application of releasing agents. However, the curing of PDMS on VeroClear resin experienced several polymerization issues, and were more difficult to release.

Importantly, the SLA mould could be used multiple times without any degradation or deformation of pattern geometries observed during the repeated PDMS curing cycles.

**Figure 4:**
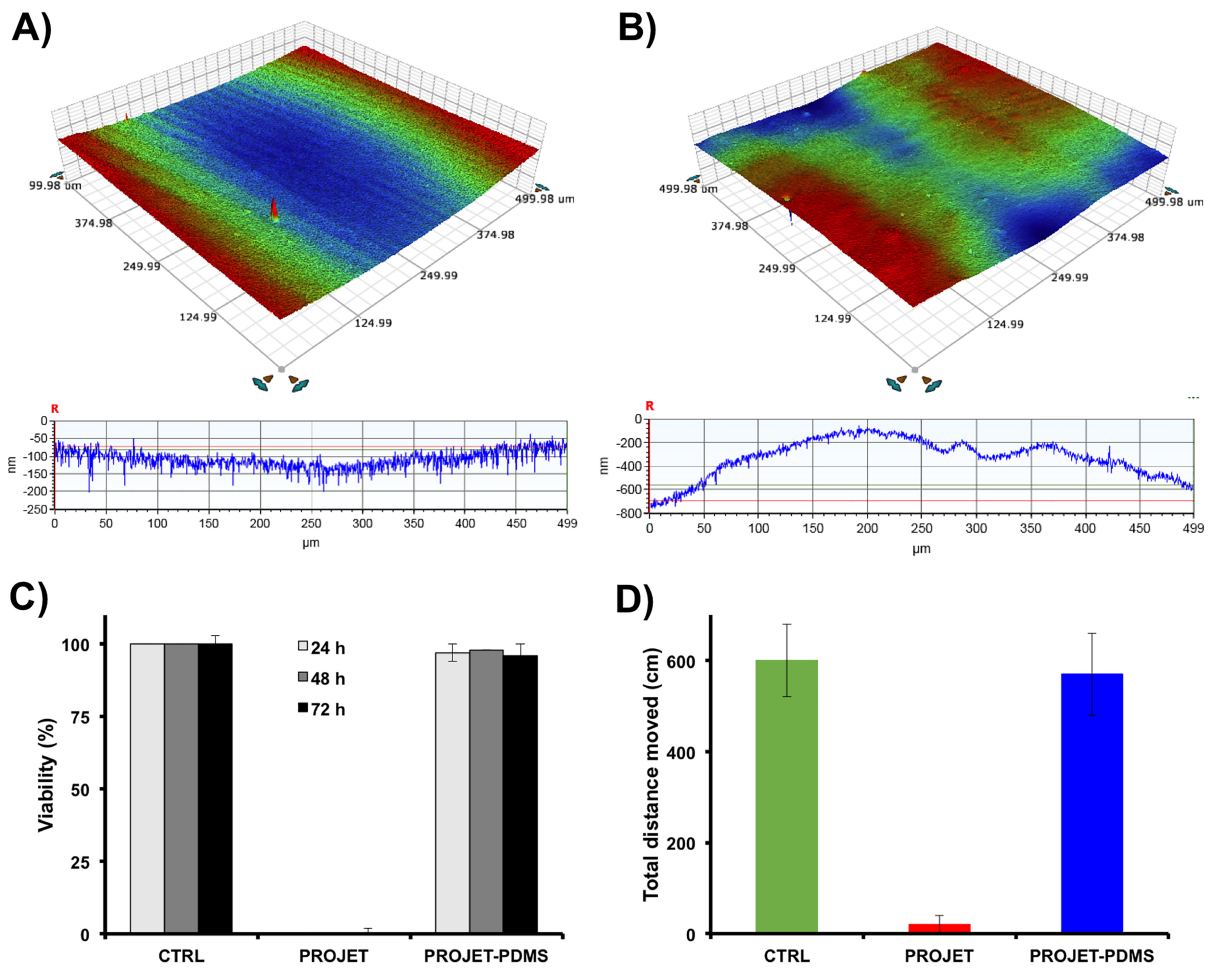
Characterization of PDMS replicas obtained from both control (CTRL) laser-cutting methods and SLA-AM (ProJet 7000 HD) positive relief moulds: (**A**) Quantitative topographic surface analysis of a representative 500 μm × 500 μm section of a millifluidic channel fabricated in PDMS from moulds made with laser cut and thermally bonded PMMA moulds; PMMA surface topography such as valleys (peak-to-peak value of 200 ± 20 nm) are thermal deformations formed during laser cutting and subsequent oven bonding. (**B**) Surface roughness renders of PDMS replicas obtained from ProJet 7000 HD printed moulds. Surface topography characteristic of SLA was peak-to-peak = 850 ± 70 nm. Measurements consisting of at least three independent samples were taken using ContourGT-I 3D optical profilometery. (**C**,**D**) Toxicity profiling of PDMS test wells casted on a mould fabricated using ProJet 7000 HD in VisiJet SL Clear resin. PDMS replicas were tested using OECD 236 Fish Embryo Acute Toxicity (FET) Assay with zebrafish (*Danio rerio*). Comparative analysis between negative control polystyrene wells (CTRL), SLA moulds printed in VisiJet Clear SL (PROJET), and wells made from moulding PDMS with Visijet Clear SL moulds (PROJET-PDMS): (C) survival of *D. rerio* larvae, and (D) behavioural responses of *D. rerio* larvae, measured as the change in total distance travelled (±S.E) after 5 min in wells. Results represent data from at least three independent experiments performed in triplicate.

Quantitative 3D topographic surface mapping using optical profilometery revealed that PDMS replicas had low surface roughness (Figure 4). Median surface roughness of PDMS replicas made from VisiJet SL Clear resin moulds was higher than the control PMMA moulds (Ra = 850 nm vs. 200 nm, respectively). The SLA moulds had had an average surface roughness of 1.7 ± 0.4 μm (taking into account both the tops of channels and bases of troughs). Consequently, following plasma treatment, PDMS replicas achieved proper hydraulic sealing to both glass and PDMS surfaces.

The PDMS millifluidic LOC produced from the SLA mould was optically transparent, thus enabling high-resolution fluorescence imaging (Figure 3).

Several studies have shown that photoinitiator and short-chain polymer residues that may leach from SLA devices exhibit a level of toxicity to test organisms [17,21,22,30,34]. This indicates that compounds remaining in the SLA-printed parts are not irreversibly trapped or adequately removed in standard manufacturer recommended post-processing protocols (e.g., solvent extraction and post-curing) [23]. This has cast doubt on the appropriateness of using SLA to produce biomicrofluidic LOC devices intended for in vitro bioassays [9,16,17,35]. However, using AM to create moulds, and the final LOC being produced by soft-lithography in PDMS side-steps these concerns.

Biocompatibility of PDMS replicas fabricated from SLA moulds was evaluated by using the well-established and very sensitive standard OECD FET test on developing *D. rerio* embryos [9,17]. The embryonic developmental stage is considered to be the most sensitive to environmental perturbations, and can be readily applied to assess impacts of any potential toxic effects of chemicals and solid phases [9,33].

The FET bioassay showed no observable toxicity of PDMS replicas fabricated using SLA moulds (Figure 4). At 24 h, all *D. rerio* embryos exposed to PDMS replicas survived without any developmental abnormalities. In contrast, at 24 h, the survival rate of embryos exposed to test parts fabricated by SLA and post-processed using standard cleaning procedures was ∼0% (data not shown). Behavioural toxicity bioassay revealed a lack of any behavioural abnormalities in zebrafish larvae exposed to PDMS replicas. In contrast, *D. rerio* larvae exposed to parts made with SLA exhibited sudden and complete paralysis. This suggests that toxic compounds did not transfer from SLA moulds to PDMS during the casting and curing process.

One of the main limitations of conventional soft lithography for millifluidic devices is the low availability of inexpensive master-mould fabrication techniques, and AM presents an attractive solution to overcoming this obstacle. Current-generation SLA technologies are generally able to print with resolutions >100 μm in the *x*–*y* dimension and >25 μm in the *z* dimension. Consequently, this technology is particularly suitable for fabricating millifluidic devices such as those designed with channel minimum cross-section dimensions ranging from 500 to 1500 μm for use with metazoan model organisms. While AM cannot match the performance and resolution of photolithography, it does permit the design and manufacture of complex geometries. SLA, rather than MJ, was shown to be a better choice for the fabrication of the millifluidic devices used in our study.

Despite some practical limitations, the significance and convenience of PDMS replica moulding and PDMS-on-glass-LOC is still high. Due to high metabolic rate, gas diffusion through PDMS layer is particularly advantageous for devices aimed at bioassays on model metazoan organisms. Moreover, the elastic properties of PDMS can alleviate mechanical damage during the loading and immobilization process of fragile embryonic stages. The replicas achieved a suitable level of pattern fidelity, with only a relatively insignificant deviation from the desired geometry of the fluidic channels. PDMS replicas also permitted bright field and fluorescent imaging at relatively high resolution. In addition, we showed that there was no transfer of toxic compounds from AM moulds to PDMS replicas.

Here we present a combinatorial fabrication approach utilizing SLA and soft lithography in PDMS as a viable method for the inexpensive production of high-definition biocompatible millifluidic LOC devices. These devices are easily adaptable for use in a range of studies using small to large metazoan model organisms. The presented technique offers the possibility of prototyping millifluidic devices and iterating multiple designs per day to support fast optimization cycles and achieve significant advancement in the definition of manufactured structures. Due to the growing availability of AM facilities, which enables the outsourced fabrication of small batches of prototypes at a very low cost, our described manufacturing method is readily accessible to any biological laboratory. In addition, issues previously observed with polymer toxicity were avoided.

## Supplementary Materials

The following are available online at http://www.mdpi.com/2072-666X/9/3/116/s1, Figure S1: Physical characteristics of semi-circular positive relief patterns fabricated using additive manufacturing processes. (A) Pattern fabricated in VisJet Clear material using the ProJet 7000 HD system; (B) PDMS replica obtained from the master depicted in (A); (C) Pattern fabricated in Vero Clear using the Objet350 Connex system; (D) PDMS replica obtained from the master depicted in (C). Dotted lines represent the designed CAD geometry superimposed on SEM images of representative cross-sections.

## Abbreviations

The following abbreviations are used in this manuscript:

LOC	Lab-on-a-Chip
AM	A dditive manufacturing
MJ	Material jetting
3D	Three-dimensional
PDMS	Poly(dimethylsiloxane)
SLA	Stereolithography
PEG-DA	poly(ethylene glycol) diacrylate
USP	United States Pharmacopeia
PMMA	Poly(methyl methacrylate)
CNC	Computer numerical control
OECDv	Organisation for Economic Co-operation and Development
CAD	Computer-assisted design
DPF	Days post-fertilisation
SEM	Scanning electron microscope
GFP	Green fluorescent protein
