# Supplementary material for: Characterization of 3D-Printed Moulds for Soft Lithography of Millifluidic Devices
Source: Micromachines (Basel). 2018 Mar 8;9(3):116. doi: 10.3390/mi9030116 (PMC6187831; doi:10.3390/mi9030116)
Supplement: Supplementary file 1 [file micromachines-09-00116-s001.pdf]

Supplementary Materials: Characterization of 3D-Printed Moulds for Soft Lithography of Millifluidic Devices

Nurul Mohd Fuad, Megan Carve, Jan Kaslin and Donald Wlodkowic

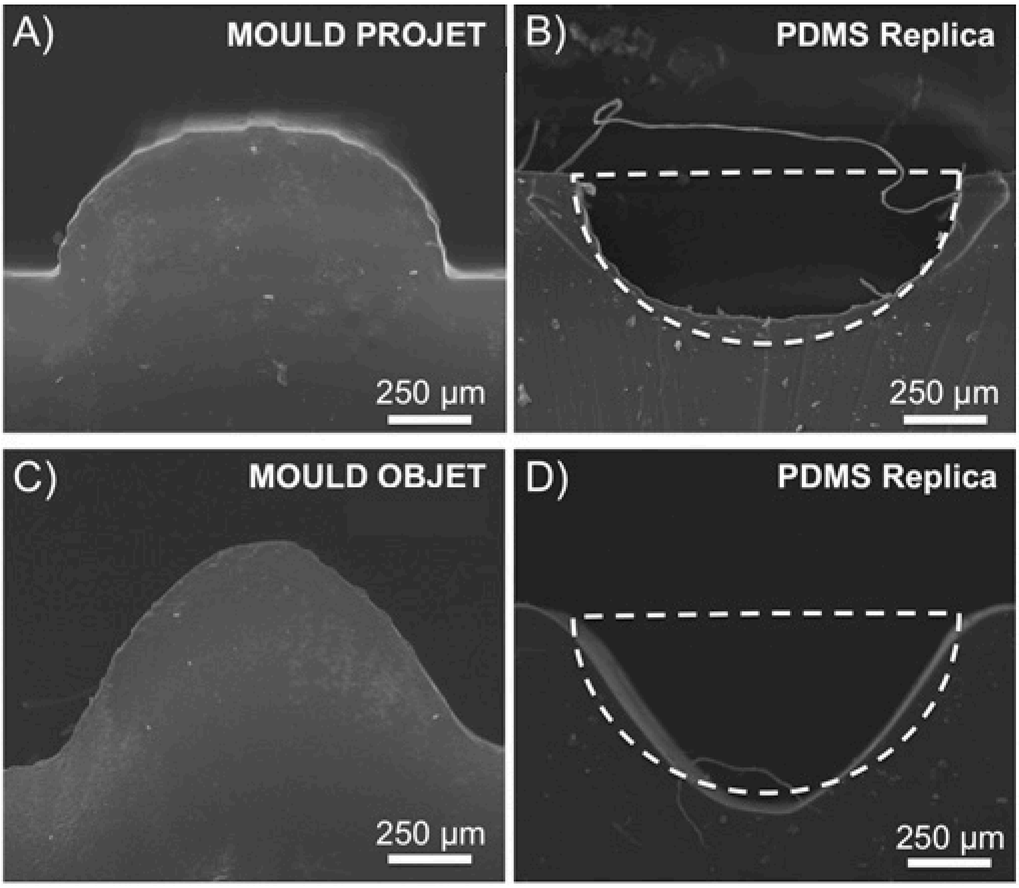

Figure S1. Physical characteristics of semi-circular positive relief patterns fabricated using additive manufacturing processes. (**A**) Pattern fabricated in VisJet Clear material using the ProJet 7000 HD system; (**B**) PDMS replica obtained from the master depicted in (A); (**C**) Pattern fabricated in Vero Clear using the Objet350 Connex system; (**D**) PDMS replica obtained from the master depicted in (C). Dotted lines represent the designed CAD geometry superimposed on SEM images of representative cross-sections.
